# Response and Resistance to Trametinib in MAP2K1-Mutant Triple-Negative Melanoma

**DOI:** 10.3390/ijms24054520

**Published:** 2023-02-24

**Authors:** Fanny Seraphine Krebs, Bianca Moura, Edoardo Missiaglia, Veronica Aedo-Lopez, Olivier Michielin, Petros Tsantoulis, Bettina Bisig, Mounir Trimech, Vincent Zoete, Krisztian Homicsko

**Affiliations:** 1 Computer-Aided Molecular Engineering, Department of Oncology UNIL-CHUV, University of Lausanne, 1015 Lausanne, Switzerland; 2 Ludwig Institute for Cancer Research, 1005 Lausanne, Switzerland; 3 Service of Medical Oncology, 1700 Fribourg, Switzerland; 4 Department of Laboratory Medicine and Pathology, Institute of Pathology, Centre Hospitalier Universitaire Vaudois, Lausanne University, 1011 Lausanne, Switzerland; 5 Molecular Modelling Group, SIB Swiss Institute of Bioinformatics, 1015 Lausanne, Switzerland; 6 Monash Medical Centre, Clayton, VIC 3168, Australia; 7 Service of Medical Oncology, Department of Oncology, Centre Hospitalier Universitaire Vaudois, 1011 Lausanne, Switzerland; 8 Centre for Personalized Oncology, Department of Oncology, Centre Hospitalier Universitaire Vaudois, 1011 Lausanne, Switzerland; 9 Swiss Cancer Center Leman, 1005 Lausanne, Switzerland; 10 Department of Oncology, Hopitaux Universitaire de Genève, 1205 Geneva, Switzerland

**Keywords:** cancer, *MAP2K1*, triple-negative melanoma, modelling, mutation

## Abstract

The development of targeted therapies for non-*BRAF* p.Val600-mutant melanomas remains a challenge. Triple wildtype (TWT) melanomas that lack mutations in *BRAF*, *NRAS*, or *NF1* form 10% of human melanomas and are heterogeneous in their genomic drivers. *MAP2K1* mutations are enriched in *BRAF*-mutant melanoma and function as an innate or adaptive resistance mechanism to BRAF inhibition. Here we report the case of a patient with TWT melanoma with a bona fide *MAP2K1* mutation without any *BRAF* mutations. We performed a structural analysis to validate that the MEK inhibitor trametinib could block this mutation. Although the patient initially responded to trametinib, he eventually progressed. The presence of a *CDKN2A* deletion prompted us to combine a CDK4/6 inhibitor, palbociclib, with trametinib but without clinical benefit. Genomic analysis at progression showed multiple novel copy number alterations. Our case illustrates the challenges of combining MEK1 and CDK4/6 inhibitors in case of resistance to MEK inhibitor monotherapy.

## 1. Introduction

Virtually all melanomas harbour MAPK pathway activation [1]. *BRAF* p.Val600 mutations are present in roughly half of melanoma patients, and therapies targeting *BRAF* mutants are one of the greatest successes in molecular oncology [2,3]. Additional MAPK pathway activating mutations can occur in *NRAS* and *NF1* genes [4]. While it has been suggested that NF1-mutant melanomas are sensitive to MEK inhibition [5], MAPK pathway targeting in *NRAS* mutant tumours has not shown consistent benefit [6]. Melanomas lacking *BRAF*, *NRAS* and *NF1* mutations form a genomically heterogeneous group of tumours, also called triple wild-type melanomas (TWT) [4]. MAPK pathway activation can also be detected in a subset of TWT melanomas. Currently, it remains unclear if and how TWT melanoma patients might respond to targeted therapies and whether combination treatments could overcome the frequent co-alterations, which limit treatment efficacy. Herein we report the case of a TWT melanoma patient with a dual *MAP2K1* mutation, highlighting the challenges of MEK inhibition monotherapy in the presence of multiple resistance mechanisms.

## 2. Results

### 2.1. Case Description

**Figure 1:**
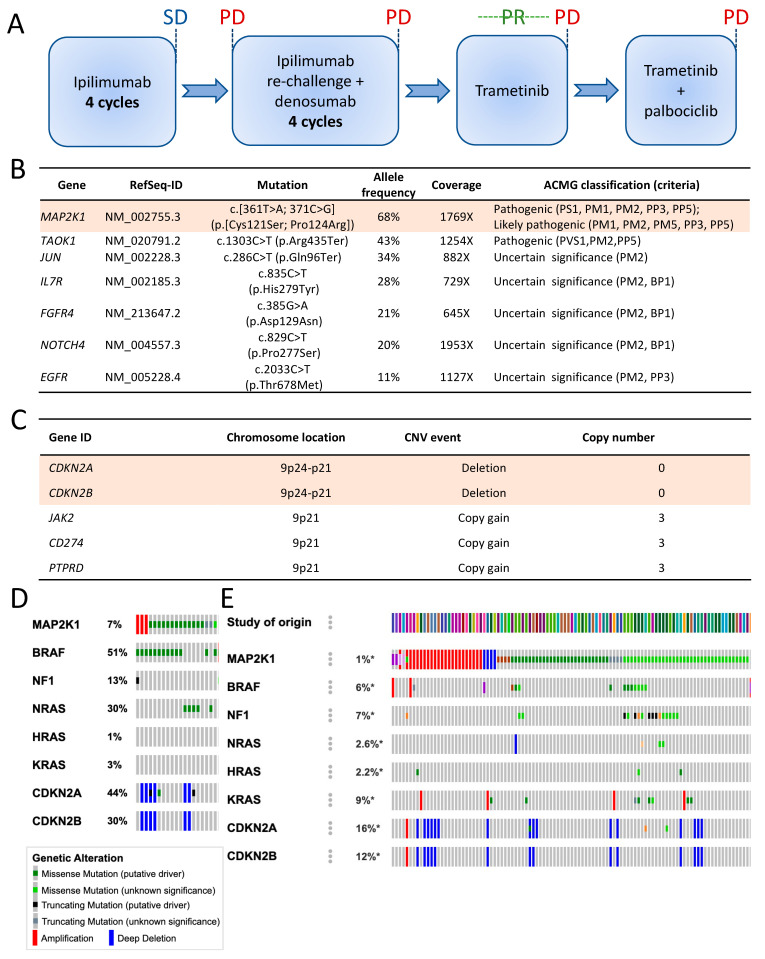
Treatment and analysis of the patient data. (**A**) Schematic figure showing the treatments received by the patient from the diagnosis of metastatic melanoma; (**B**) List of mutations detected by NGS of pre-treatment biopsy; (**C**) Selection of copy number variations detected by next-generation sequencing of pre-treatment biopsy; (**D**) Oncoprint analysis of MAP2K1-mutant melanoma patients of the TCGA melanoma (SKCM) cohort for other MAPK genes and *CDKN2A/B*; (**E**) Oncoprint analysis of MAP2K1 mutant cancer patients of the TCGA cohorts except for melanoma for other MAPK genes and *CDKN2A/B*. * is a symbol used for nonsense and frameshift mutations.

We present the case of a 55-year-old Caucasian male, initially in good general health. The primary tumour was detected in 2003 as a nodular melanoma, Breslow 1.52 mm, Clark IV in the right scapular region, and resected with a 2 cm safety margin. Sentinel lymph node (SLN) histological examination revealed multiple scattered micrometastases, and the patient benefited from a completion lymph node dissection (CLND), showing no further metastatic lesions. The final staging of the primary melanoma was pT2a pN1a cM0 (Stage IIIA as per AJCC 7th edition). Eleven years later, the patient presented with a solitary lung lesion that was surgically removed by segmentectomy. The patient’s disease further progressed in 2016, requiring systemic therapy. The patient also developed chronic renal failure secondary to focal segmental glomerulosclerosis (FSGS) and received a kidney transplant, independent of the melanoma diagnosis. The kidney transplantation limited immune therapy options to CTLA4 inhibition (ipilimumab) and excluded PD-1 inhibitors, which could lead to acute organ rejection. The patient started ipilimumab in June 2016 and received four 3 mg/kg doses with a best objective response of stable disease (SD) according to RECIST1.1 (Figure 1A). Ipilimumab administration induced an autoimmune nephritis, reversible by immune-suppressive corticosteroid therapies. The patient completely recovered renal functions and agreed to a re-challenge with ipilimumab combined with denosumab for bone metastases. The patient’s disease failed to respond to the re-challenge and progressed again in February 2018, prompting next-generation sequencing (NGS) analysis for alternative targeted therapies.

### 2.2. Genomic Analysis

After an initial hotspot NGS analysis had shown the absence of mutations in *BRAF*, *NRAS* and *KIT* genes, we performed an extended NGS using an in-house developed panel covering the full-coding sequences of 394 cancer-associated genes. We achieved mean sequencing coverages of 1128X and 390X for tumour and normal genomic DNA extracted from peripheral blood mononuclear cells (PBMCs), respectively. The estimated tumour content was 70%. We identified two *MAP2K1* mutations, located in cis (on the same allele), both at 68% variant allele frequencies: p.Cys121Ser known as activating [7,8,9,10,11,12,13], and p.Pro124Arg. A truncating mutation in exon 13 of *TAOK1* gene was classified as pathogenic, and five additional mutations in other genes were classified as variants of uncertain significance (VUS), according to the American College of Medical Genetics and Genomics (ACMG) guidelines (Figure 1B). The tumour mutation burden (TMB) was calculated at 5.4 non-synonymous mutations/megabase, which is relatively low for melanomas. Additionally, copy number analysis found focal, likely homozygous deletions of *CDKN2A* and *CDKN2B* (Figure 1C), in conjunction with other large-scale, non-focal copy number variations (CNVs). We estimated a low large-scale state transition (LST) score, a marker of homologous recombination deficiency (HRD).

In melanoma, *MAP2K1* mutations are usually associated with other MAPK pathway mutations, including in *BRAF* and *NRAS*. Indeed, an analysis of The Cancer Genome Atlas (TCGA) database of the melanoma cohort (SKCM) identified only one patient (1/287 patients) with *MAP2K1* mutant melanoma without other MAPK pathway mutations (Figure 1D), underscoring the rarity of the patient’s genetic constellation. The analysis of all TCGA datasets, excluding melanoma, showed that patients with *MAP2K1* mutations in tumours were largely devoid of mutations in other MAPK pathway genes (Figure 1E).

### 2.3. Molecular Modelling Analysis of MAP2K1 Mutations

Although mutation *MAP2K1* p.Cys121Ser was previously identified as pathogenic, we were concerned that its association with p.Pro124Arg, whose functional impact is less well understood, might influence or prevent response to MEK1 inhibitors, such as trametinib. In addition, the vast majority of reads supported the haplotype with the two mutations showing an allele frequency close to the tumour content, suggesting a clonal origin of such combination. Therefore, we performed structural analysis of the MAP2K1 protein to better understand the potential impact of the two mutations combined. 

**Figure 2:**
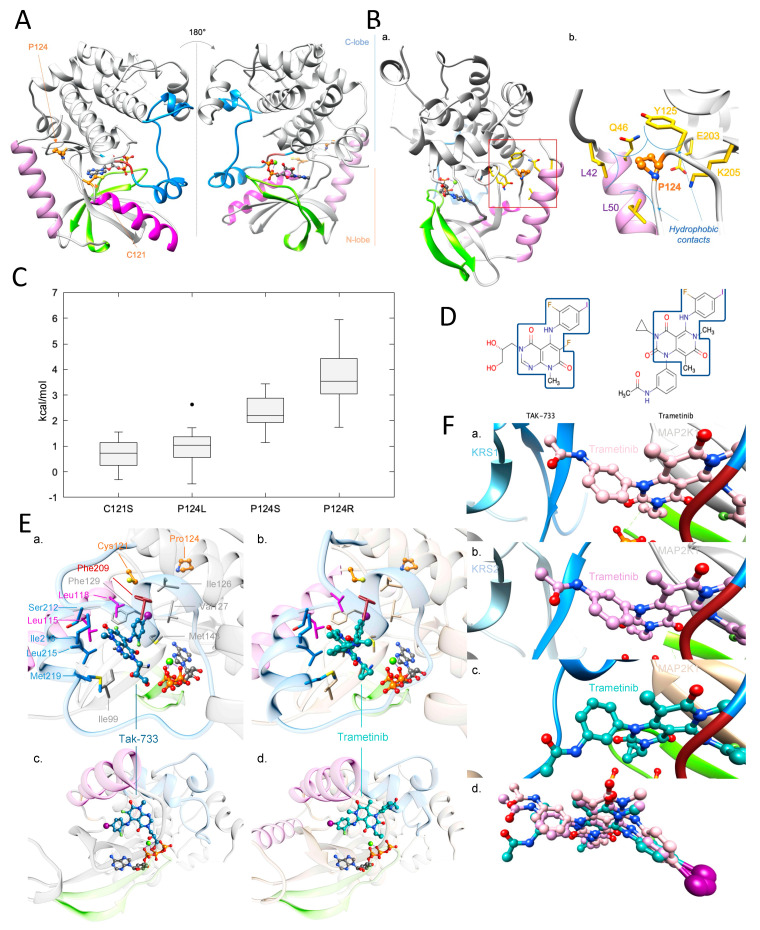
Molecular modelling analysis: (**A**) Representation of the MAP2K1 kinase structure in the presence of the ADP and the Mg^2+^ in ball-&-stick. The P-loop is coloured in green, the C-helix in magenta, the A-loop in blue, the Helix-A in pink and the DFG motif in dark red. Cys121 and Pro124 are in orange ball-&-stick (pdb: 3eqi) [17]; (**B**) MAP2K1 Pro124 environment. (**a**) Kinase structure; (**b**) Zoom on MAP2K1 Pro124, represented in orange ball-&-stick, and its neighbour residues in a sphere of 5Å, represented in sticks and coloured in yellow. MAP2K1 kinase structure is in the presence of the ADP and the Mg^2+^ in ball-&-stick. The P-loop is coloured in green, the C-helix in magenta, the A-loop in blue, the helix-A in pink and the DFG motif in dark red. (pdb: 3eqi) [17]; (**C**) Folding free energy distribution calculated with FoldX, in kcal/mol, for *MAP2K1* p.Cys121Ser, p.Pro124Leu, p.Pro124Ser and p.Pro124Arg. The center line of the boxes represents the median and the whiskers extent from the ends of the box to the most distant point. The value outside of the limits is drawn individually. *MAP2K1* p.Cys121Ser is mentioned for information and is discussed in the Supplementary Materials; (**D**) TAK-733 and trametinib MEK inhibitors. The common molecular moieties between these two molecules is framed; (**E**) Structural analysis. (**a**,**c**): Structure of Tak-733 bound to MAP2K1 in presence of Mg^2+^ and NADPH (pdb: 3pp1) [18]; (**b**,**d**): Model of trametinib bound to MAP2K1 in the presence of Mg^2+^ and NADPH. Residues interacting with Tak-733 or trametinib are in sticks, ligands in ball-&-stick and the protein in ribbons. The key regions P-loop, Activation loop, DFG motif and C-helix are coloured as follows: green, blue, dark red and magenta. Cys121 and Pro124 are represented in orange ball-&-stick; (**F**) Trametinib binding. (**a**) Trametinib binding in MAP2K1:KRS1complex in the presence of Mg^2+^ and ANP (pdb: 7jux) [19]; (**b**) Trametinib binding in MAP2K1:KRS2 complex in the presence of Mg^2+^ and ANP (pdb: 7jur) [19]; (**c**) Trametinib binding in a MAP2K1 docked model in the presence of Mg^2+^ and ATP; (**d**) superimposition of the three binding modes. Ligands are in ball-&-stick, protein in ribbons.

Protein kinases, such as MAP2K1, catalyse the transfer of a phosphate group from ATP to specific protein substrates. The MAP2K1 kinase is involved in many cellular processes such as cell proliferation, development and differentiation. It is activated by RAF1, which is activated upstream with RAS, by extracellular signals, such as a MAP2K1/MEK1 dual-specific protein kinase. Kinase common architecture contains N- and C-lobes connected by a flexible hinge, seat of the enzymatic reaction including ATP/ADP binding site. The N-lobe gathers Helix-A, five-stranded β-sheets and C-helix (Figure 2A). Helix-A is specific to the MAP2K1 kinase. It is situated in the early section of the N-lobe and allows conformation-dependent autoregulation of the protein activity [14]. In the inactive conformation, it interacts with the N-lobe, unlike in the active one. The C-lobe comprises helices around a hydrophobic core, and contains the A-loop, including the highly conserved motif Asp-Phe-Gly (DFG), important for enzyme activity and ligand binding (Figure 2A) [15,16]. The *MAP2K1* p.Cys121Ser mutation is known in the literature as activating, including in melanoma [7,8,9,10,11,12,13]. Its analysis is provided in the Supplementary Figure S1.

Several *MAP2K1* p.Pro124 mutations were previously identified. The *MAP2K1* p.Pro124Ser/Leu/Gln mutations alone are predicted to be activating in several cancers, including melanoma. *MAP2K1* p.Pro124Ser/Leu shows resistance to PLX4720, a pan-RAF inhibitor. *MAP2K1* p.Pro124Ser/Gln presents moderate resistance to Dabrafenib [9,12,20,21,22], a BRAF V600 inhibitor. Pro124 is in the N-lobe bend, following C-helix. Proline is often involved in bends because of its cyclic structure, constraining the protein backbone. In inactive conformation, Pro124 interacts via hydrophobic interactions with Helix-A residues: Leu42, Gln46, Leu50 (Figure 2B). Pro124 also participates in hydrophobic interactions with Tyr125. These interactions induce a tight hydrophobic cluster, allowing Helix-A protein activity regulation. 

Arginine is large and positively charged, whereas proline is small and uncharged. Due to its size and Helix-A proximity, *MAP2K1* p.Pro124Arg is predicted to generate a substantial steric clash, leading to the destabilisation of the inactive conformation in favour of the active one. With Helix-A repelled, the mutant will be oriented toward solvent but will not impact the kinase binding site. To address time constraints in the context of the patient’s emergency, FoldX [23] was used to estimate the impact of the *MAP2K1* p.Pro124Arg mutation on 3D structures, including Helix-A (Figure 2C). The FoldX folding free-energy distribution ranged from 1.7 to 5.9 kcal/mol, with a median at 3.5 kcal/mol, which indicates that the mutant is expected to have a significant adverse impact on protein folding. The same calculations were performed for *MAP2K1* p.Pro124Ser/Leu mutations. Estimated folding free-energies increased with the polarity of the mutant, as p.Pro124Leu values were the lowest, followed by p.Pro124Ser and p.Pro124Arg. This observation is consistent with our previous structural analysis (i.e., Pro124 contributes to the stabilization of the region via hydrophobic interactions with its environment). The impact of p.Pro124Arg was determined experimentally after our molecular modelling analysis and found to enhance the protein activity, in agreement with our prediction [24].

### 2.4. Investigation of a Potential MAP2K1 Inhibitor and Mutations

Trametinib is an established clinically approved MEK inhibitor. At the time of our analysis, no experimental structure of trametinib with *MAP2K1* existed. Therefore, we used structural bioinformatic methods to predict its binding mode to the *MAP2K1* double mutant. Since similar molecules bind similarly to similar targets [25], the experimental structure of TAK-733 (pdb: 3pp1 [18], see Supplementary Materials; Figure 2D), which has the highest FP2 similarity to trametinib among all crystallised MAP2K1 ligands, was used to predict the trametinib binding mode.

The 2-fluoro-4-iodoaniline group of the trametinib inhibitor was predicted to bind similarly to the one present in TAK-733 in the 3D structure 3pp1 [18]. Both moieties made similar hydrophobic and polar interactions (Figure 2E). Trametinib-substituted pyridopyrimidine and 2-fluoro-4iodoaniline groups were expected to make: (i) hydrophobic interactions with Leu115, Leu118 from the C-helix; Ile99, Ile126, Val127, Gly128, Phe129, Ile141, Met143 from the N-lobe; Phe209, Gly210 from the DFG motif; Ser212, Leu215, Ile216, Met219 from the A-loop; (ii) hydrogen bonds with Lys97, Ser212 backbones. The main differences between TAK-733 and trametinib came from pyridopyrimidine substitutions. Trametinib was substituted by cyclopropane and acetanilide moieties that were predicted to reinforce hydrophobic contacts with the A-loop by interacting with Met219. They may also have stabilised the P-loop through potential interactions with Gly79. The acetanilide group reached the C-ter part of the A-loop, allowing potential hydrogen bonding with Arg234 and hydrophobic interactions with Met230. However, the acetamide function may have had a local steric impact, especially with Arg189 and Asp190. Finally, p.Cys121Ser and p.Pro124Arg mutations were not oriented toward the predicted trametinib binding site (Figure 2E) and should not have significantly decreased its binding affinity. Based on this analysis, trametinib was suggested as a potential treatment for the patient.

Recently, M. Khan et al. published experimental structures of MAP2K1:KRS1 and MAP2K1:KRS2 [19], both with trametinib binding to the allosteric inhibitor site, as predicted in our model (Figure 2F). The RMSDs between their MAP2K1:KRS1 and MAP2K1:KRS2 trametinib conformations and our model were only 0.76 Å and 0.71 Å, respectively, showing that our model was accurate (See Supplementary Materials for more information).

### 2.5. Clinical Course

**Figure 3:**
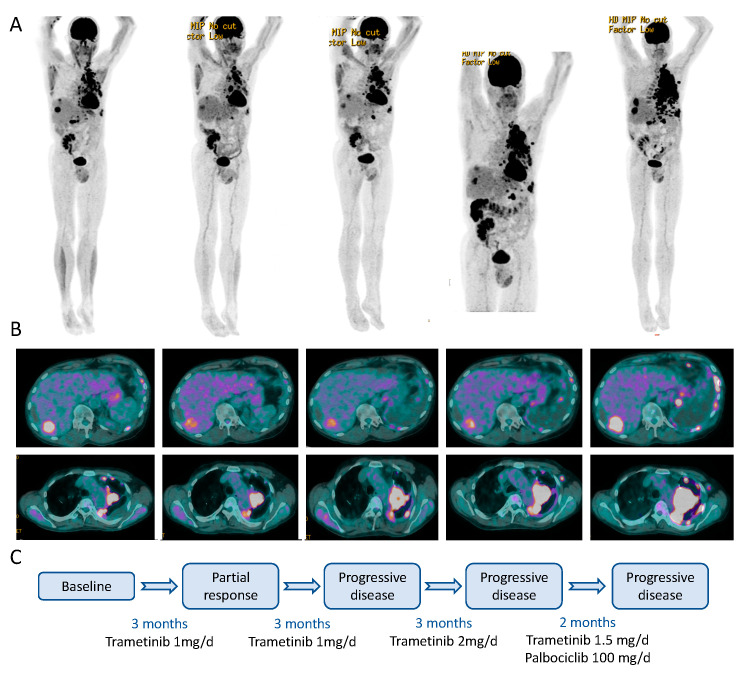
PET images and treatment of the patient. (**A**) Whole body PET images, showing tracer accumulation in tumour lesions; (**B**) Cross section of the PET images, showing responses of a liver lesion (upper panel) and para-aortic tumour masses during the therapeutic interventions; (**C**) Schematic time course of the MEK inhibitor treatments and responses.

Considering the *MAP2K1* activation as the unique driver oncogene of the patient’s tumour, we initiated therapy with trametinib with a full dose of 2 mg/day. However, the dose had to be reduced to 1 mg/day due to grade three toxicities (fatigue and rash). After two months of treatment, we detected a good partial response (PR) (Figure 3). After an additional three months, we detected an increase in tumour volumes from the maximum response, though still below the initial tumour volumes. Therefore, we increased trametinib doses back to 2 mg/day. Despite the increased trametinib dosage with better tolerance than at first exposure, the patient’s disease continued to progress. In the absence of viable therapeutic options, we hypothesised that the deletion of the *CDKN2A* locus could be a mechanism of resistance to MEK inhibition. We therefore proposed the off-label use of the CDK4/6 inhibitor palbociclib in combination with trametinib. After obtaining approval from the patient’s insurance, he was started on palbociclib 125 mg/day for 21 days every 28 days with continuous trametinib at 1 mg/day. However, the palbociclib dosage had to be adjusted to 100 mg and eventually 75 mg/day due to recurrent grade three fatigue. After two months of combined palbociclib and trametinib therapy, the patient’s disease continued to progress, and trametinib and palbociclib were discontinued (Figure 3). The patient then received additional chemotherapy, and his disease was again re-challenged with ipilimumab. He ultimately succumbed to melanoma in 2019.

### 2.6. Genomic Changes in Response to MEK Inhibition

**Figure 4:**
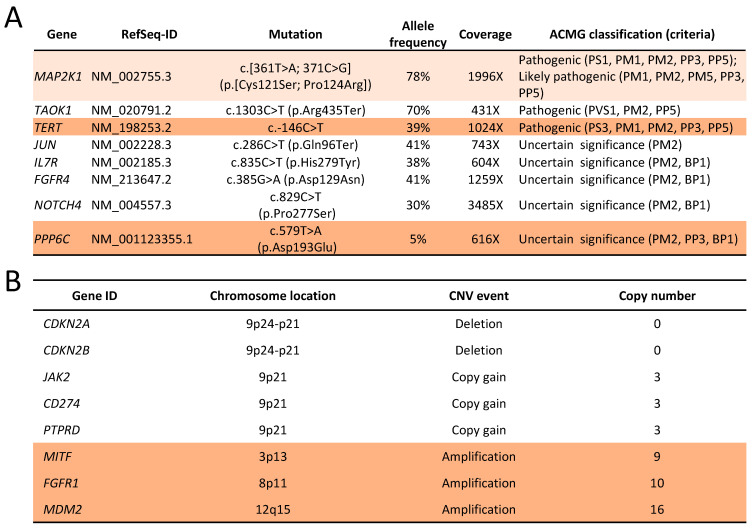
(**A**) List of mutations detected by next-generation sequencing in a tumour biopsy after failure of MEK inhibition alone; (**B**) Selection of copy number variations detected by next-generation sequencing in a tumour biopsy after failure of MEK inhibition alone.

We performed a new NGS analysis on a tumour sample obtained from a pleural biopsy after trametinib monotherapy failed. The assay showed the persistence of the original MAP2K1 mutations and the absence of any new *MAP2K1* gatekeeper mutation that would have prevented inhibition by trametinib (Figure 4A). We detected the loss of a class 3 *EFGR* mutation in a minor clone and the appearance of mutations in *TERT* promoter and *PPP6C*. The TMB was similar to the baseline. In contrast with the relative lack of changes in the mutation profiles, we detected many novel CNVs (Figure 4B). Notably, the tumour developed high-level amplification of *MDM2*, *FGFR1* and *MITF* while maintaining the homozygous loss of *CDKN2A* and *CDKN2B*. We found no evidence of homologous recombination deficiency.

## 3. Discussion

We found it unusual that the patient harboured two cis *MAP2K1* mutations, p.Cys121Ser and p.Pro124Arg, shared by most cancer cells. Our structural analysis validated that even the dual mutant was amenable to *MAP2K1* inhibition. This study shows the power of structural analysis to complement genomic analyses in guiding treatment selection for precision medicine. *MAP2K1* has been described as an active therapeutic target in Langerhans cell histiocytosis (LCH) [8,26]. Trametinib monotherapy has shown exceptional levels of tumour responses, frequently achieving complete responses in LCH [27]. In melanoma, *MAP2K1* mutations are typically detected along with other MAPK-activating mutations, such as those in *BRAF* and *NRAS*, and serve as resistance mechanisms to BRAF and dual BRAF/MEK inhibitor therapies [28]. In contrast, our analysis showed that in non-melanoma solid tumours, *MAP2K1* mutations typically exist without additional *BRAF*, *RAS*, or *NF1* which could also be sensitive to MEK inhibitors. Our patient’s tumour showed a clinically significant partial tumour response. However, after five months the treatment failed, which is suggestive of adaptive resistance mechanisms. CDK4/6 inhibitors, such as palbociclib or abemaciclib, have now been approved for the treatment of metastatic, hormone-receptor-positive breast cancer [29], irrespective of genomic alterations. To date, no genomic alteration or biomarker has been shown to predict sensitivity to CDK4/6 inhibitors [30]. *CCND1* amplification has been suggested as a potential predictor of CDK4/6 inhibitor benefit, although recent work by the NCI-MACTH consortium has shown an absence of correlation [31]. Previous preclinical work also suggested that CDK4/6 inhibitors could overcome resistance to the MAPK pathway, specifically to MEK inhibition [32]. In the absence of other therapeutic options, we started the trametinib/palbociclib combination. 

However, the dual therapy failed only after 2 months. Genetic analysis of the biopsy after trametinib failure excluded the presence of novel *MAP2K1* gatekeeper mutations. One could hypothesise that, unlike receptor tyrosine kinases (EGFR, ALK), intracellular MAPK pathway inhibitors of BRAF or NRAS do not induce secondary gatekeeper mutations. In contrast to mutations, the numerous copy number alterations could be the source of resistance. The *MDM2* amplification could explain the combination treatment’s lack of efficacy. In absence of *TP53* mutation, *MDM2* amplification could inhibit wildtype *TP53* functions, including cell cycle control. Hence, *TP53* dysregulation could prevent the control of the cell cycle by CDK4/6 inhibition. Combined treatment with blockers of TP53/MDM2 interaction, such as nutlins, could have been proposed to the patient [33]. However, we could not find any clinical trial that would have accepted our patient with kidney transplantation. Alternatively, *FGFR1* amplification could also lead to resistance to MAPK pathway inhibition [34]. *FGFR1* amplification would lead to the PI3K pathway activation [35], limiting MAPK pathway inhibition [36]. We could not obtain a pan-FGFR inhibitor to co-target with trametinib and palbociclib. 

Finally, we could only consecutively administer trametinib and the trametinib/palbociclib combination. Ideally, the dual genomic alterations of *MAP2K1* and *CDKN2A* could have been co-targeted from the beginning, which might have resulted in a deeper and lasting tumour response. However, it is currently impossible to obtain inhibitor combination for rare, off-label indications, despite the absence of therapeutic alternatives.

This case presents strong evidence supporting *MAP2K1* mutation identification for TWT melanoma and solid tumours. Our analyses underscore the need to better define rational combination therapies for genomic co-alterations and to consider combination therapies before monotherapies. Analysis of treatment failures could highlight the rapid tumour adaptation, opening potential avenues for combination therapies. This study underscores the utility of genomic analysis in TWT melanoma as well as the usefulness of molecular modelling analysis to assess potential impacts of uncharacterised mutations on protein activity and drug resistance in the context of personalised medicine.

## 4. Materials and Methods

Amino acid sequences were retrieved from the UniProt database [37] and sequence alignments were performed using MUSCLE v3.8.31 (EMBL-EBI, Cambridge, UK) [38]. The visualization software UCSF Chimera v1.13.1 (University of California San Francisco, San Franscico, CA, USA) [39] was used for analysis of the structures and sequence alignments. The Foldx5 software (CRG-EMBL-VIB consortium) [23], was used for the estimation of the impact of a mutation on protein structures via the PositionScan command line. The Protein Data Bank [40] ID of the structures used for this study are: 3zlw [41], 3zls [41], 5bx0 [42], 5eym [43], 5hze (to be published), 3zly [42], 3zlx [42], 3zm4 [42], 3eqc [17], 3eqg [17], 3eqf [17], 3sls [44], 3w8q (to be published), 3eqd [17], 3eqi [17], 3eqh [17], 6u2g [45], 5yt3 (to be published), 3pp1 [18], 7jux [19], 7jur [19]. Only structures in which both residues Cys121 and Pro124 and the Helix-A are resolved were used for the analysis. Openbabel v2.4.1 (University of Pittsburgh, Pittsburgh, PA, USA) was used for the molecular similarity calculations [46] using FP2 molecular fingerprints. The Tanimoto coefficient was used to quantify the molecular similarity, and ranges from 0 for totally different molecules to 1 for identical compounds. The predicted binding mode of trametinib on MAP2K1 was calculated using the Attracting Cavities [47] docking tool applied to the protein 3D structure 3pp1, after removing the TAK-733 ligand, using default parameters. To relax potential constraints and obtain the final docked structure, the calculated binding mode was minimised via 1000 steps of SD algorithm followed by 1000 steps of ABNR algorithm using the CHARMM [48] v40 program (University of Harvard, Cambridge, MA, USA), the CHARMM36 [49] force field to describe the protein and SwissParam parameters for the ligand [50]. The solvation effect was taken into account using the GB-MV2 implicit solvation model [51].

## Data Availability

Patient data are unavailable due to privacy restrictions. Molecular modelling data used for this study are publicly available for academic research. The corresponding references are provided in the Section 4.

## Supplementary Materials

The following supporting information can be downloaded at: https://www.mdpi.com/article/10.3390/ijms24054520/s1.
