# Supplementary material for: Response and Resistance to Trametinib in MAP2K1-Mutant Triple-Negative Melanoma
Source: Int J Mol Sci. 2023 Feb 24;24(5):4520. doi: 10.3390/ijms24054520 (PMC10003177; doi:10.3390/ijms24054520)
Supplement: Supplementary file 1 [file ijms-24-04520-s001.zip › ijms-2201571-supplementary.pdf]

Supplementary materials

1. Molecular Modelling analysis

1.1. MAP2K1 p.Cys121Ser

Cys121 is in the beginning of the N-lobe and is structurally close to the activation loop (207-241), which are critical regions for the enzymatic activity (Figure 1a SI). Cys121 is partially buried in a hydrophobic cluster involving the following residues: i) Ile126 from the N-lobe; ii) Val117, Leu118 from the C-helix, iii) Phe209 from the DFG motif; iv) Tyr179, Leu180, Ile184, His184 from the C-lobe (Figure 1b SI). Thus, Cys121 plays an important role in the structural stabilization of its environment. Serine properties differ from those of cysteine, as it is polar and its side chain cannot participate in hydrophobic interactions, as does that of cysteine. Consequently, the mutant *MAP2K1* p.Cys121Ser will not be able to maintain wild-type interactions, leading to a less stable region. Mutation *MAP2K1* p.Cys121Ser is depicted in the literature as an activating mutation in lung cancer, melanoma, and other diseases and to confer resistance to combined RAF and MEK inhibitors vemurafenib and selumetinib, respectively [7]. The instability generated by the mutant could trigger a conformational change causing the protein activation and the modification of binding affinity of ligands such as inhibitors.

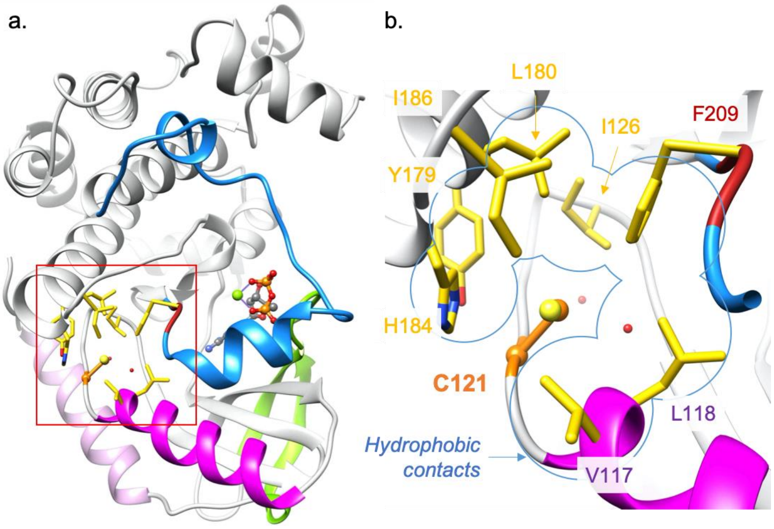

Figure S1. Environment of the position MAP2K1 p.Cys121. a) Structure of the kinase domain; b) Zoom in on MAP2K1 p.Cys121shown as orange ball-&-stick, and its neighbouring residues in a 5Å-radius sphere, shown as sticks and coloured in yellow. The potential hydrophobic contacts are shown as blue spheres. MAP2K1 kinase structure is in presence of ADP and Mg^{2+} in ball-&-stick. The P-loop is coloured green, the C-helix magenta, the A-loop blue, the helix-A pink and the DFG motif dark red. (PDB: 3eqi) [4].

The *MAP2K1* p.Cys121Ser folding free energy calculation was done using FoldX [23] as a comparison with *MAP2K1* p.Arg124 mutations and will not be discussed since the effect of the mutation was already characterized at the time of our analysis. By comparison, for the same wild-type structures, *MAP2K1* p. Cys121Ser distribution ranges from -0.3 to 1.5 kcal/mol, with a median at 0.7 kcal/mol. These results highly suggest that p.Pro124Arg structural impact on the inactive conformation of the protein is more important than p.Cys121Ser one.

2. Sequence analysis

The sequence alignment of reviewed sequences of *MAP2K1* orthologs with Human showed that the entire region of the mutants is highly conserved, like Helix-A (Figure S2), which does not allow estimating the potential significance of a particular residue. In this situation, the sequence analysis of the orthologs does not help us to predict the impact of

mutations. Interestingly, the analysis of human paralogs of *MAP2K1* shows more diversity. Indeed, both positions *MAP2K1* p.Cys121 and Pro124 are only both conserved in *MAP2K2* at positions 125 and 128 respectively, which shares a sequence identity with MAP2K1 of 81%, while for *MAP2K7* and *MAP2K6*, it oscillates between 32 and 37%, respectively. *MAP2K2* Cys125, which corresponds to *MAP2K1* Cys121, leads to a gain of function and to resistance effect, like in MAP2K1 [3]. At the time of our analysis, no mutation impacting *MAP2K2* Pro128 was known. But the recent publication of Hanrahan et al.[24], showed that *MAP2K1* p.Pro128Leu is leading to a potential gain of function, similarly to what is observed for the *MAP2K1* corresponding residue [12.]

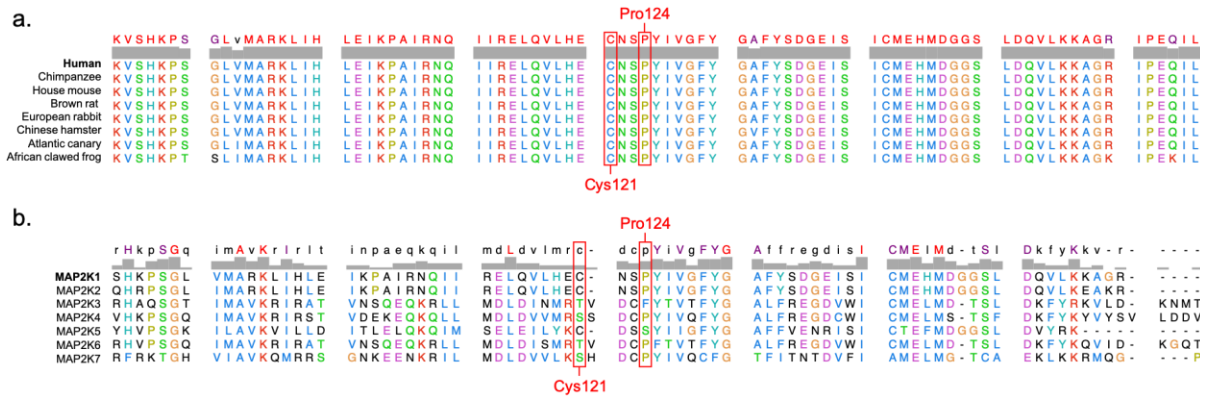

Figure S2. Multiple alignment sequences. a. Zoom in on the residues of the human MAP2K1 p.Cys121 and MAP2K1 p.Pro124 from the alignment of reviewed sequences of MAP2K1 human with orthologs; b. Zoom in on the residues of the human MAP2K1 p.Cys121 and MAP2K1 p.Pro124 from the alignment of reviewed sequences of human MAP2K1 with paralogs.

3. Structural similarity of molecules

The structural similarity of one molecule to another was calculated using the Tanimoto coefficient, based on the FP2 fingerprints of the molecules. The higher the Tanimoto coefficient, the more similar the couple of molecules, with a maximum of 1 when the two molecules are identical. Conversely, a low Tanimoto coefficient will be observed for non-similar couple of molecules. Among all MAP2K1 ligands with published experimental structure, TAK-733 has the highest Tanimoto coefficient to trametinib and was selected to continue the analysis (i.e., Tanimoto coefficient: 0.53).

4. Trametinib binding site. Comparison of the predicted model and a recent publication

Recently, trametinib potential mechanism of action has been described [19], when M Khan *et al*. published the first crystallographic structures of complexes MAP2K1:KRS1 and MAP2K1:KRS2, both co-crystallized in presence of trametinib, which binds to the allosteric inhibitor site, close to ATP, as predicted in our docked model. The overall root-mean-square-deviations (RMSD) between their MAP2K1:KRS1 and MAP2K1:KRS2 structures and the one we used for our model are only of 0.76 Å and 0.71 Å respectively. The main difference comes from the residues 218 to 229, at the interface with KSR1/2 in the A-loop. During our analysis, this interaction was not known. If we compare our predicted pose of trametinib and the one present in the experimental structures MAP2K1:KRS1 and MAP2K1:KRS2, we obtain RMSD values of 2.36 Å and 2.24 Å for the global molecule and of 0.90 Å and 0.49 Å for the core, respectively. This RMSD variance comes from the acetanilide group which, in the crystallographic structures, interacts with an extended conformation of the MAP2K1 A-loop via a polar interaction involving the carbonyl group of the acetamide function and the Arg234 and via a hydrophobic interaction with a residue from the KSR1/2 C-lobe, Ala825 and Pro878 respectively.

References:

1. Wagle N, Emery C, Berger MF, Davis MJ, Sawyer A, Pochanard P, Kehoe SM, Johannessen CM, Macconaill LE, Hahn WC, Meyerson M, Garraway LA. Dissecting therapeutic resistance to RAF inhibition in melanoma by tumor genomic profiling. J Clin Oncol. 2011 Aug 1;29(22):3085-96. doi: 10.1200/JCO.2010.33.2312. Epub 2011 Mar 7. PMID: 21383288; PMCID: PMC3157968; Brown NA, Furtado LV, Betz BL, Kiel MJ, Weigelin HC, Lim MS, Elenitoba-Johnson KS. High prevalence of somatic MAP2K1 mutations in BRAF V600E-negative Langerhans cell histiocytosis. Blood. 2014 Sep 4;124(10):1655-8. doi: 10.1182/blood-2014-05-577361. Epub 2014 Jun 30. PMID: 24982505; Kinoshita-Kikuta E, Kinoshita E, Ueda S, Ino Y, Kimura Y, Hirano H, Koike T. Increase in constitutively active MEK1 species by introduction of MEK1 mutations identified in cancers. Biochim Biophys Acta Proteins Proteom. 2019 Jan;1867(1):62-70. doi: 10.1016/j.bbapap.2018.05.004. Epub 2018 May 9. PMID: 29753091; Nelson DS, van Halteren A, Quispel WT, van den Bos C, Bovée JV, Patel B, Badalian-Very G, van Hummelen P, Ducar M, Lin L, MacConaill LE, Egeler RM, Rollins BJ. MAP2K1 and MAP3K1 mutations in Langerhans cell histiocytosis. Genes Chromosomes Cancer. 2015 Jun;54(6):361-8. doi: 10.1002/gcc.22247. Epub 2015 Mar 31. PMID: 25899310; Emery CM, Vijayendran KG, Zipser MC, Sawyer AM, Niu L, Kim JJ, Hatton C, Chopra R, Oberholzer PA, Karpova MB, MacConaill LE, Zhang J, Gray NS, Sellers WR, Dummer R, Garraway LA. MEK1 mutations confer resistance to MEK and B-RAF inhibition. Proc Natl Acad Sci U S A. 2009 Dec 1;106(48):20411-6. doi: 10.1073/pnas.0905833106. Epub 2009 Nov 13. PMID: 19915144; PMCID: PMC2777185; Narita Y, Okamoto K, Kawada MI, Takase K, Minoshima Y, Kodama K, Iwata M, Miyamoto N, Sawada K. Novel ATP-competitive MEK inhibitor E6201 is effective against vemurafenib-resistant melanoma harboring the MEK1-C121S mutation in a preclinical model. Mol Cancer Ther. 2014 Apr;13(4):823-32. doi: 10.1158/1535-7163.MCT-13-0667. Epub 2014 Jan 21. PMID: 24448821.
2. Delgado J, Radusky LG, Cianferoni D, Serrano L. FoldX 5.0: working with RNA, small molecules and a new graphical interface. Bioinformatics. 2019 Oct 15;35(20):4168-4169. doi: 10.1093/bioinformatics/btz184. PMID: 30874800; PMCID: PMC6792092.
3. Long GV, Fung C, Menzies AM, Pupo GM, Carlino MS, Hyman J, Shahheydari H, Tembe V, Thompson JF, Saw RP, Howle J, Hayward NK, Johansson P, Scolyer RA, Kefford RF, Rizos H. Increased MAPK reactivation in early resistance to dabrafenib/trametinib combination therapy of BRAF-mutant metastatic melanoma. Nat Commun. 2014 Dec 2;5:5694. doi: 10.1038/ncomms6694. PMID: 25452114; Hanrahan AJ, Sylvester BE, Chang MT, Elzein A, Gao J, Han W, Liu Y, Xu D, Gao SP, Gorelick AN, Jones AM, Kiliti AJ, Nissan MH, Nimura CA, Poteshman AN, Yao Z, Gao Y, Hu W, Wise HC, Gavrila EI, Shoushtari AN, Tiwari S, Viale A, Abdel-Wahab O, Merghoub T, Berger MF, Rosen N, Taylor BS, Solit DB. Leveraging Systematic Functional Analysis to Benchmark an *In Silico* Framework Distinguishes Driver from Passenger MEK Mutants in Cancer. Cancer Res. 2020 Oct 1;80(19):4233-4243. doi: 10.1158/0008-5472.CAN-20-0865. Epub 2020 Jul 8. PMID: 32641410; PMCID: PMC7541597.
4. Hanrahan AJ, Sylvester BE, Chang MT, Elzein A, Gao J, Han W, Liu Y, Xu D, Gao SP, Gorelick AN, Jones AM, Kiliti AJ, Nissan MH, Nimura CA, Poteshman AN, Yao Z, Gao Y, Hu W, Wise HC, Gavrila EI, Shoushtari AN, Tiwari S, Viale A, Abdel-Wahab O, Merghoub T, Berger MF, Rosen N, Taylor BS, Solit DB. Leveraging Systematic Functional Analysis to Benchmark an In Silico Framework Distinguishes Driver from Passenger MEK Mutants in Cancer. Cancer Res. 2020 Oct 1;80(19):4233-4243. doi: 10.1158/0008-5472.CAN-20-0865. Epub 2020 Jul 8. PMID: 32641410; PMCID: PMC7541597.
5. Emery CM, Vijayendran KG, Zipser MC, Sawyer AM, Niu L, Kim JJ, Hatton C, Chopra R, Oberholzer PA, Karpova MB, MacConaill LE, Zhang J, Gray NS, Sellers WR, Dummer R, Garraway LA. MEK1 mutations confer resistance to MEK and B-RAF inhibition. Proc Natl Acad Sci U S A. 2009 Dec 1;106(48):20411-6. doi: 10.1073/pnas.0905833106. Epub 2009 Nov 13. PMID: 19915144; PMCID: PMC2777185.
6. Khan ZM, Real AM, Marsiglia WM, Chow A, Duffy ME, Yerabolu JR, Scopton AP, Dar AC. Structural basis for the action of the drug trametinib at KSR-bound MEK. Nature. 2020 Dec;588(7838):509-514. doi: 10.1038/s41586-020-2760-4. Epub 2020 Sep 14. PMID: 32927473; PMCID: PMC7746607.
